# Supplementary material for: Measuring the frequency chirp of extreme-ultraviolet free-electron laser pulses by transient absorption spectroscopy
Source: Nat Commun. 2021 Jan 28;12:643. doi: 10.1038/s41467-020-20846-1 (PMC7843717; doi:10.1038/s41467-020-20846-1)
Supplement: Supplementary file 1 — Supplementary Information [file 41467_2020_20846_MOESM1_ESM.pdf]

Supplementary Information for "Measuring the frequency chirp of extreme-ultraviolet free-electron laser pulses by transient absorption spectroscopy"

T. Ding *et al.*

Supplementary Note 1: Accelerator settings and performance

The measurements have been performed at the free-electron laser in Hamburg, FLASH[1]. With the accelerator settings of 1.2-kA peak current, an emittance of 1 mm-mrad, an electron energy spread of ΔE = 0.2 MeV at 515 MeV electron beam energy, and a beta function of 10 m, we get a (natural) photon spectral bandwidth of about $(\Delta\omega/\omega)_{\mathrm{FEL}}$ = 0.4% (private communication with M.V. Yurkov). In fact, the spectral bandwidth is a slow function of the electron beam parameters. Based on the experimentally measured FEL spectra (see Fig. 3a in the main text), the XUV spectral bandwidth, $\Delta\omega/\omega = 1.6\%$, was significantly larger than the natural bandwidth, there is strong evidence of a chirp. This finding is supported by the electron beam compression settings that were not set to full compression, which was done on purpose in order to achieve a broader photon bandwidth for transient absorption measurements. Unfortunately, no dispersive electron phase-space measurements were performed to quantify the RF-induced chirp of the electron bunch.

In addition, longitudinal space-charge fields, induced by a current spike, can induce an energy chirp on the electron bunch[2]. However, since the measured bunch current profile [transverse deflecting RF structure (TDS) measurement with a 120-fs FWHM electron bunch, see Supplementary Fig. 1] does not show a current spike, the influence of the space-charge effects seems low. We therefore assume that the broad-bandwidth accelerator tuning, utilizing a not fully compressed electron bunch, is the main explanation for the observation of a significant chirp of the FEL photons which is discussed in the main text.

Supplementary Note 2: Order-of-magnitude estimation of nonlinear chirp contributions

We approximate the measured dispersion function $\tau(\omega)$ [cf. equation (1) of the main text] by third-order Taylor-series expansion about the center photon energy $\omega_{\mathrm{L}} \approx 50.3$ eV. The measured absolute FEL spectral bandwidth at half-width at half maximum (HWHM), $\Delta\omega_{\mathrm{HWHM}} = |\omega_{\mathrm{min/max}} - \omega_{\mathrm{L}}|$ (with the spectral wing positions ω_{min} and ω_{max}), is about 0.4 eV, or 0.6 rad/fs, and allows for an order-of-magnitude estimation of the individual Taylor expansion coefficients D_n of the spectral phase $\Phi(\omega)$ [cf. equation (2) of the main text]. Given the polynomial coefficients extracted from the fit to the measured data for moderately low FEL-pulse energies between 35-45 µJ (see Fig. 4 of the main text), i.e., $D_2 = 52$ fs²/rad, $D_3 = -15$ fs³/rad², $D_4 = -133$ fs⁴/rad³ the relative weights of the second-, third-, and fourth-order terms to $\Phi(\omega)$ are about 9.6, 0.56, and 0.76 rad at the HWHM position of the spectrum (calculated via $|D_n \times (\Delta\omega_{\mathrm{HWHM}})^n/n!|$). This estimation demonstrates that the lowest second-order linear chirp clearly dominates the trend of the nonlinear spectral phase, while the impact of D_4 is still minor despite its comparatively large numerical value even for a moderately low pulse energy.

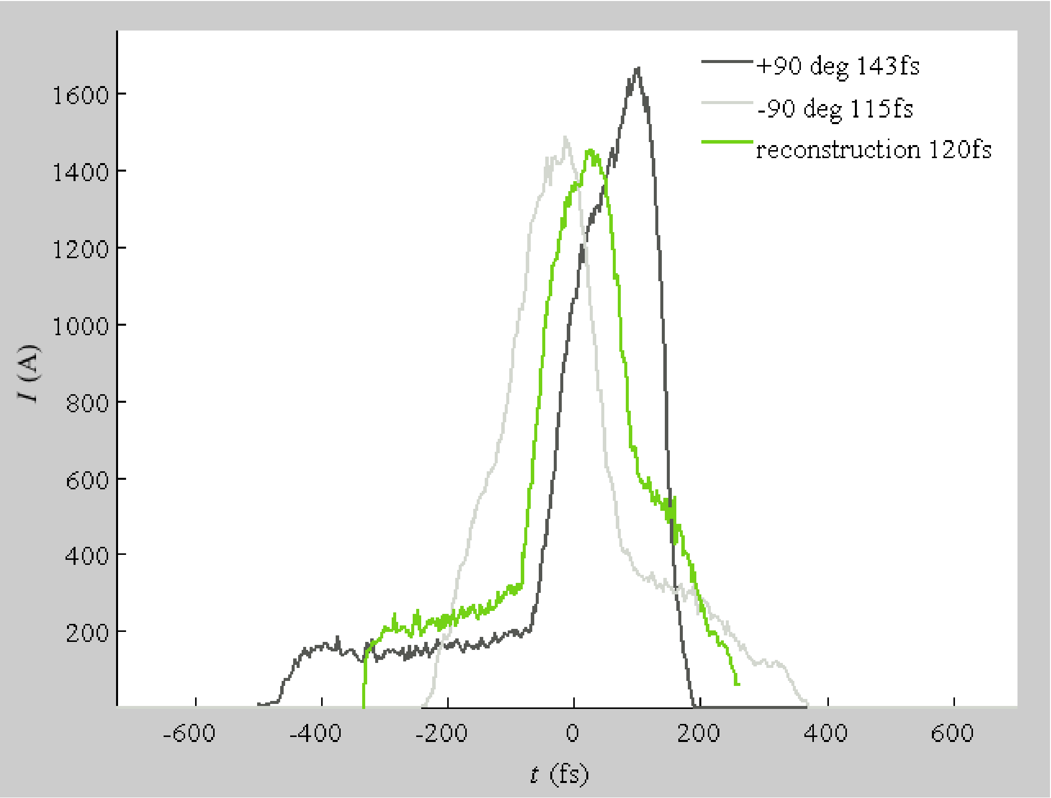

Supplementary Figure 1: Electron bunch length measurement using a transverse deflecting structure (TDS). The green line displays the TDS measurement of the electron-bunch profile. The reconstructed width (FWHM) is about 120 fs.

Supplementary References

1. Ackermann, W. *et al.* Operation of a free-electron laser from the extreme ultraviolet to the water window. *Nat. Photonics* **1**, 336–342 (2007).
2. Dohlus, M. *et al.* Start-to-end simulations of SASE FEL at the TESLA Test Facility, phase 1. *Nucl. Instruments Methods Phys. Res. Sect. A Accel. Spectrometers, Detect. Assoc. Equip.* **530**, 217–233 (2004).
